# A six-month crossover chemoprevention clinical trial of tea in smokers and non-smokers: methodological issues in a feasibility study

**DOI:** 10.1186/1472-6882-12-96

**Published:** 2012-07-16

**Authors:** Chiranjeev Dash, Fung-Lung Chung, Joy Ann Phillips Rohan, Emily Greenspan, Patrick D Christopher, Kepher Makambi, Yukihiko Hara, Kenneth Newkirk, Bruce Davidson, Lucile L Adams-Campbell

**Affiliations:** 1 Department of Oncology, Lombardi Comprehensive Cancer Center, Georgetown University, Washington, D.C, USA; 2 Department of Otolaryngology, Georgetown University, Washington, D.C, USA; 3 College of Dentistry, Howard University, Washington, D.C, USA; 4 Tea Solutions, Hara Office Inc, Tokyo, Japan; 5 Lombardi Comprehensive Cancer Center, Georgetown University Medical Center, Research Building, E501. 3970 Reservoir Rd, N.W, Washington, D.C, USA

## Abstract

**Background:**

Chemoprevention crossover trials of tea can be more efficient than parallel designs but the attrition and compliance rates with such trials are unknown.

**Methods:**

Attrition (dropouts) and compliance with treatment were assessed in a 25-week randomized, placebo controlled, crossover, feasibility clinical trial of four tea treatments to investigate the effect of tea on oral cancer biomarkers. Each treatment lasted 4 weeks with 2 weeks of washout in between. Participants were 32 smokers and 33 non-smokers without any evidence of premalignant oral lesions. The interventions consisted of packets of green tea, black tea, caffeinated water, or placebo. Participants were assigned to each treatment for four weeks, and were instructed to drink five packets per day while on the treatment. Dropout from the trial and compliance (consumption of ≥ 85% of the prescribed treatment packets) are the main outcome measures reported.

**Results:**

There was a high rate of dropout (51%) from the study, and the rates were significantly higher among smokers (64%) than non-smokers (36%). Among participants who completed the study the rate of compliance was 72%. The highest rates of dropouts occurred between the first and second treatment visits in both smokers (38% dropout) and non-smokers (18% dropout). Throughout the study smokers were more likely to dropout than non-smokers. Black tea treatment was associated with the highest rates of dropout among smokers (37%), but was associated with the lowest rate of dropout among non-smokers (4%).

**Conclusions:**

In a study conducted to test the feasibility of a four-treatment crossover tea trial, a high rate of dropout among smokers and non-smokers was observed. Multi-arm crossover tea trials might pose a higher burden on participants and research is needed to improve adherence and treatment compliance in such trials.

**Trial registration number:**

ISRCTN70410203

## Background

Crossover clinical trials, where participants serve as their own control, afford higher efficiency in both power and cost over parallel designs and have recently seen increasing popularity in evaluating complementary and alternative medicine (CAM) therapies [1]. The efficiency and validity of a crossover design, however, is dependent on participants’ adherence to treatment and limited attrition during the study [2]. This is especially true in trials with more than two arms where a greater burden is placed on the participants. Knowledge of predictors associated with compliance is therefore important to increase and maintain compliance during crossover studies.

The role of tea, both green and black, in the prevention of cancer is supported by evidence from a large number of studies conducted in cell culture and in animal bioassays [3], but results of epidemiologic studies have been inconsistent [4]. Although this discrepancy between laboratory and epidemiologic studies underscores the need for chemoprevention trials of tea, only a limited number of clinical trials have been conducted. Two small trials in patients with premalignant oral lesions demonstrated that the administration of tea results in a reduced risk of oral cancer [5,6]. However, it is not known whether tea has a role in the primary prevention of oral cancer, and whether the effect of tea on oral cells might be different in smokers than non-smokers. It is also not known whether a multi-arm crossover treatment of tea treatments will be feasible and whether participants will comply with the treatments over the comparatively long course of such a study. A feasibility four-arm crossover trial was conducted to evaluate the effect of green tea, black tea, caffeinated water, and placebo on cancer biomarkers in oral cells of smokers and non-smokers without premalignant lesions, and data on patient participation and compliance in the study were analyzed.

Although reports on short-term crossover trials to assess the efficacy of green/black tea for prevention of various chronic diseases have been published in the last few years, rates of attrition and compliance, and factors affecting such rates have rarely been reported [7-10]. Here, dropout and compliance rates in a feasibility crossover trial of tea are reported, and demographic and behavioral factors associated with study dropout and compliance are examined.

## Methods

A 25-week, double-blind, four-arm crossover, feasibility randomized trial of the effect of tea on cancer biomarkers in oral cells of smokers and non-smokers was conducted at the Lombardi Comprehensive Cancer Center, Georgetown University Medical Center between 2007 and 2009. Men and women between 18–60 years of age who did not habitually drink more than two servings per day of tea, coffee or caffeinated beverages or alcoholic beverages were invited to participate in the study. Participants reporting habitual consumption of less than 2 cups/day of tea or coffee were eligible to be part of the study. Subjects with oral lesions, allergies to tea/coffee, cancer or under advisement not to take excessive caffeine were ineligible for the study. Recruitment strategies, utilizing flyers posted at various common areas in the Georgetown University campus, aimed at equal numbers of smoking (at least 10 cigarettes/day) and non-smoking participants. Both participants and study staff were blinded to the assigned treatment. The trial protocol and study procedures were approved by the Georgetown University institutional review board.

**Figure 1:**
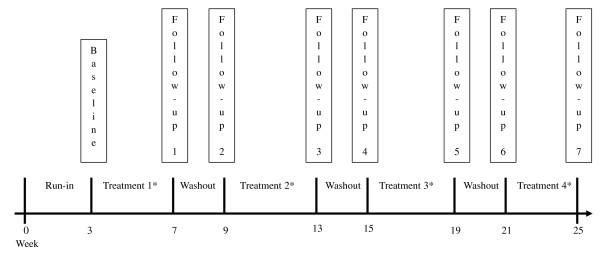
**Study design schema of the 25-week crossover tea study.** Legend: * Treatments: Green tea, Black tea, Caffeinated water, and Placebo.

After providing informed consent, participants underwent a three week run-in prior to starting the regimen. During the run-in period the participants were asked not to consume tea, coffee or other caffeine-containing beverages. Participants were not assigned placebo during the run-in period and no attempt was made to assess compliance during the run-in period. Smokers and non-smokers were then randomized to a crossover treatment regimen of green tea, black tea, caffeinated water, and placebo (containing all elements of the tea beverages except the tea). The randomization was stratified by smoking status. Participants were assigned four weeks of each treatment with two weeks of washout between each of the four crossover treatments (Figure 1). At baseline and each follow-up visit the participants completed study questionnaires and oral cells were collected for biomarker measurements. The participants underwent clinical examination and oral health evaluation at each visit to ensure no adverse effects from the treatments. The frequency, interval, and duration of the treatments were determined based on previous pilot trials of tea [5,6].

The treatments were provided in standardized coded packets manufactured by Mitsui Norin Co., Ltd., Tokyo, Japan. There was 400 mg of tea powder in each of the packets containing green/black tea. Each caffeinated water packet contained caffeine equivalent to that in a cup of green tea. The treatments were designed to make the color and taste of the four treatments as similar to each other as possible to preserve participant blinding. Formal studies were not conducted to determine whether the tea treatments were actually indistinguishable from each other in taste. The bioactive and chemopreventive components of green and black tea are primarily attributed to polyphenolic compounds in tea. While catechins, specifically Epigallocatechingallate (EGCG), are the most abundant polyphenols in green tea, theaflavins are more abundant in black tea. The composition of polyphenols in each packet of tea is shown in Table 1. Participants were instructed to drink 5 packets per day and were asked to dissolve the contents of the treatment packet in a cup of cold water prior to consumption. Participants were instructed to follow a specified protocol including drinking two cups in the morning, 2 cups in the afternoon, and 1 cup after dinner. A standard method of drinking was prescribed whereby participants were instructed to drink by mouthfuls, holding each mouthful for 30 seconds to 1 minute to facilitate the uptake of tea in the oral cells.

**Table 1:** Composition of tea packets in the crossover tea study

**Component**	**Green tea**	**Black tea**
	**(%, g/100 g)**	**(%, g/100 g)**
**Flavan-3-ols**		
(+)-Gallocatechin	2.5	0.2
(−)-Epigallocatechin	8.0	0.4
(+)-Catechin	1.2	0.9
(−)-Epigallocatechingallate	12.1	0.7
(−)-Epicatechin	3.0	0.6
(+)-Gallocatechingallate	1.1	0.2
(−)-Epicatechingallate	3.9	0.6
(+)-Catechingallate	0.2	<0.1
**Theaflavins**		
Theaflavin	-	0.3
Theaflavin 3-*O*-gallate	-	0.5
Theaflavin 3’-*O*-gallate	-	0.3
Theaflavin 3,3’-di-*O*-gallate	-	0.4
**Caffeine**	5.4	7.1
**Gallic acid**	0.2	0.9

During the course of the study each participant was assigned to the four tea treatments with the order of treatments determined by the randomization schedule. A total of 140 packets were provided for each treatment of the crossover regimen – five packets per day for four weeks. After four weeks, participants were instructed to return the empty packets which were then tallied and recorded by one of the trial coordinators. Compliance was measured by the number of empty packets returned to the trial coordinator after each crossover treatment. The proportion of packets returned out of the 140 packets distributed for each phase of the crossover was expressed as a percentage. Participants who returned at least 85% of empty packets for each treatment were classified as “compliant”. Participants who did not complete all four crossover treatments were classified as dropouts from the study.

Data on demographics, smoking habits, and usual intake of tea, coffee, carbonated beverages, alcohol, and fruit and vegetables were recorded on questionnaires administered at baseline. Body mass index (BMI) of participants was also calculated from height and weight measured at baseline.

*Statistical analysis:* Analyses were conducted to determine factors associated with treatment compliance and study dropout. Association of dropout/compliance status with participant characteristics were based on univariate generalized linear models for the continuous variables, and the *chi-square* test for the categorical variables. For comparisons with sparse categorical data (N < 5) we used the Fisher’s exact test for tests of significance.

## Results

**Figure 2:**
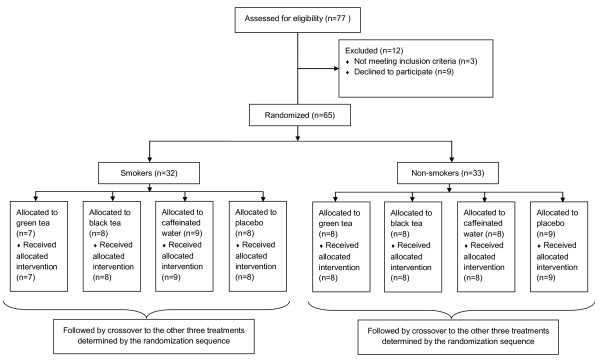
Participant flow diagram.

Thirty two current smokers (≥10 cigarettes/day) and 33 non-smokers were randomized to the four crossover treatments. The participation rate for the trial was 76% for current smokers, and 94% for non-smokers. The flow of participants along the trial is detailed in Figure 2**.** At the end of the trial period, the attrition rate across the four crossover treatments was 51% (33 participants). Of those who completed the study, 28% (9 participants) returned less than 85% of the packets for at least one treatment and were classified as “non-compliant”.

Characteristics of the participants by attrition status are presented in Table 2. Dropouts were more likely to be African American and current smokers, and less likely to be Asian and regular tea/coffee drinkers as compared to participants who completed the study. However, only the difference between smokers and non-smokers was statistically significant (Table 2).

**Table 2:** Participant characteristics by treatment completion* in the crossover tea study

**Characteristic**	**Completed (N = 32)**	**Dropped out (N = 33)**
	**(%)**	**(%)**
Age, mean (SD)	33 (12)	33 (12)
Male	44	42
Race/Ethnicity		
White	44	46
African-American	9	36
Hispanic	6	6
Asian	38	6
Other	3	6
Smoking status^**a**^		
Current	34	64
Former	6	-
Never	60	36
Regular alcohol intake	25	21
Regular tea intake	41	33
Regular coffee intake	52	41
Regular carbonated beverages intake	32	49
Fruit/vegetable servings/day, mean (SD)	2.4 (1.3)	2.7 (1.7)
BMI, mean (SD)	24.1 (3.6)	24.2 (5.8)

^**a**^*P* value <0.05 for comparison between “completed” and “dropped out”.

^**b**^ 0.05 ≤ *P* value < 0.10 for comparison between “completed” and “dropped out”.

^**c**^ 0.10 ≤ *P* value < 0.15 for comparison between “completed” and “dropped out”.

* Treatment completion is defined as completion of all four crossover treatments.

Regular intake <2 servings per day. Participants reporting ≥2 servings per day consumption of tea, coffee, carbonated beverages, or alcohol were excluded from the trial.

Among those who did not dropout, participants who were non-compliant (returned <85% empty packets for at least one treatment) were more likely to be younger, female, never smokers, and have normal BMI as compared to participants who were compliant (Table 3). They were also less likely to be regular tea and coffee drinkers than compliant participants. However, none of the differences were statistically significant.

**Table 3:** Participant characteristics by treatment compliance* among participants who completed the crossover tea study

**Characteristic**	**Compliant (N = 23)**	**Non-compliant (N = 9)**
	**(%)**	**(%)**
Age, mean (SD)	35 (11)	28 (10)
Male	48	33
Race/Ethnicity		
White	44	44
African-American	13	-
Hispanic	4	11
Asian	35	44
Other	4	-
Smoking status		
Current	39	22
Former	4	11
Never	57	67
Regular alcohol intake	22	33
Regular tea intake	44	33
Regular coffee intake	59	33
Regular carbonated beverages intake	36	22
Fruit/vegetable servings/day, mean (SD)	2.4 (1.4)	2.4 (1.0)
BMI, mean (SD)	24.9 (3.6)	22.1 (2.8)

^**a**^*P* value <0.05 for comparison between “compliant” and “non-compliant”.

^**b**^ 0.05 ≤ *P* value < 0.10 for comparison between “compliant” and “non-compliant”.

^**c**^ 0.10 ≤ *P* value < 0.15 for comparison between “compliant” and “non-compliant”.

* Treatment non-compliance defined as consumption of <85% of the packets provided for at least one of the four treatments.

Regular intake <2 servings per day. Participants reporting ≥2 servings per day consumption of tea, coffee, carbonated beverages, or alcohol were excluded from the trial.

Attrition data classified by treatment number (1 through 4) and treatment type (green tea, black tea, caffeinated water, and placebo) for current smokers and non-smokers are shown in Table 4. Attrition was highest between the first and second treatments (between weeks 3 and 7) among both smokers and non-smokers. However, dropout rates were higher for smokers as compared to non-smokers for all treatments/visits. For example, 38% of smokers dropped out between the first and second treatment visits as compared to 18% of the non-smokers (Table 4).

**Table 4:** Treatment completion* among current cigarette smokers and non-smokers in the crossover tea study

	**Smokers**			**Non-smokers**		
	**Total**	**Completed**	**Dropped out**	**Total**	**Completed**	**Dropped out**
	**(n)**	**n (%)**	**n (%)**	**(n)**	**n (%)**	**n (%)**
***Treatment number***						
1^**b**^	32	20 (62)	12 (38)	33	27 (82)	6 (18)
2	20	16 (80)	4 (20)	27	24 (89)	3 (11)
3	16	13 (81)	3 (19)	24	22 (92)	2 (8)
4	13	11 (85)	2 (15)	22	21 (95)	1 (5)
***Treatment type***						
Green tea	18	13 (72)	5 (28)	27	24 (89)	3 (11)
Black tea^**a**^	22	14 (63)	8 (37)	24	23 (96)	1 (4)
Caffeinated water	20	17 (85)	3 (15)	28	24 (86)	4 (14)
Placebo	21	16 (77)	5 (23)	27	21 (85)	6 (15)

^**a**^*P* value <0.05 for difference in proportions between smokers and non-smokers.

^**b**^ 0.05 ≤ *P* value < 0.10 for difference in proportions between smokers and non-smokers.

^**c**^ 0.10 ≤ *P* value < 0.15 for difference in proportions between smokers and non-smokers.

* Treatment completion is defined as completion of all four crossover treatments.

Treatment number represents the number of the assigned crossover treatment. For e.g., treatment number 1 is the first assigned treatment after the run-in period.

In addition, participants dropping out of the study between treatments 1 and 2 were classified as “early dropouts” and those that dropped out after receiving treatment 2 packets as “late dropouts”. Baseline characteristics between early and late dropouts were compared (Table 5). Compared to late dropouts, early dropouts were more likely to be African-American and less likely to report regular consumption of tea or coffee (0.10 ≤ P <0.15 for these comparisons). Among those who completed the treatment, compliance was slightly higher among smokers as compared to non-smokers but the difference was not statistically significant (data not shown).

**Table 5:** Participant characteristics by drop-out time* among those who dropped out of the crossover tea study

**Characteristic**	**Early drop-out **	**Late drop-out**
	**(N = 18)**	**(N = 15)**
		**(%)**	**(%)**
Age, mean (SD)	34 (12)	32 (12)
Male	39	47
Race/Ethnicity ^**c**^		
White	33	60
African-American	45	27
Other	22	13
Smoking status		
Current	67	60
Former	-	-
Never	33	40
Regular alcohol intake	33	7
Regular tea intake^, **c**^	22	47
Regular coffee intake^, **c**^	33	50
Regular carbonated beverages intake	50	47
Fruit/vegetable servings/day, mean (SD)	2.8 (1.7)	2.7 (1.7)
BMI, mean (SD)	24.1 (7.1)	24.33 (3.9)

^**a**^*P* value <0.05 for comparison between “early drop-out” and “late drop-out”.

^**b**^ 0.05 ≤ *P* value < 0.10 for comparison between “early drop-out” and “late drop-out”.

^**c**^ 0.10 ≤ *P* value < 0.15 for comparison between “early drop-out” and “late drop-out”.

* Drop-out time categorized as “early” for those who dropped out between the first and second treatment visits, and “late” for those who dropped out after the second treatment visit.

Regular intake <2 servings per day. Participants reporting ≥2 servings per day consumption of tea, coffee, carbonated beverages, or alcohol were excluded from the trial.

Association of a particular treatment (green tea/black tea/caffeinated water/placebo) with attrition/compliance was also examined (Table 4). The dropout rates for green tea, black tea, and placebo were higher for smokers than non-smokers. However, the difference was statistically significant only for black tea (Table 4). The rates for caffeinated water were similar between smokers and non-smokers. Current smokers were most likely to drop out when the assigned treatment was black tea as compared to other treatments. This was contrary to the experience among non-smokers who were least likely to drop out when on black tea treatment (Table 4). However, among the non-smokers who completed the treatments, non-compliance was higher for black tea as compared to the other treatments (data not shown).

There were no adverse effects reported by participants in the study except for those referring to unpleasant taste and teeth staining reported by some participants who dropped out of the study. Although dropouts were not re-contacted to determine the reason for dropout, some participants did call the study coordinators to inform them of their decision to drop out of the study. In these cases, the participants were asked the reason for dropping out of the study. Of the 33 participants who dropped out of the trial, 4 (12%) provided a reason for leaving the study. Of these, 2 complained of unpleasant/bitter taste of the treatment (one patient was on green tea and one on black tea at the time of dropping out), one participant complained of teeth staining (on black tea), and one participant complained of both unpleasant taste and teeth staining (on black tea).

## Discussion

In a 25-week crossover tea trial a very high attrition rate, especially among smokers, was observed. The dropout rate was highest between the first and second tea treatments. Black tea treatment was associated with the highest rate of attrition among smokers. Demographic and behavioral factors other than smoking, although suggestive, were not statistically significantly associated with attrition or compliance in our study.

Few previous crossover studies have explicitly reported on participant attrition and compliance in tea trials and none of them have reported a dropout/non-adherence rate as high as seen in this study [7-12]. None of those studies had four crossover treatments, and all were significantly shorter in duration compared to our study. In addition, none of the studies had a smoking population as large as this study.

Research on the association of smoking and attrition in clinical trials is primarily based on smoking cessation studies, which have shown that pre-inclusion (during screening or intake evaluations) attrition is generally higher (in the 30-50% range) as compared to post-inclusion (intervention or post-intervention follow-up) attrition which varies from less than 10% to 50% [13-15]. These rates are comparable to the rates seen in the present study. Rates of attrition for both smokers and non-smokers in this study were higher for the first treatment as compared to the subsequent crossover treatments.

Black tea, and to a lesser extent, green tea were associated with the highest rates of attrition among smokers but not among non-smokers. Five smokers and three non-smokers in the current trial complained of an unpleasant/bitter taste while on either the green tea or black tea treatment. However, only one of the smokers reported not complying as a result of taste, whereas the others complied with the treatments. Although smoking is common in tea houses in some cultures in Asia and Africa, and there is no research to suggest that smoking alters the taste of tea, it is possible that the polyphenols, theaflavins or other substances in black tea interact with smoking to cause a bitter taste, and might reduce the tolerance of smokers to tea treatments. Efforts to minimize attrition in future CAM studies of tea should focus primarily on smokers.

In addition to smoking, other possible reasons for this relatively high rate of non-adherence could be related to the frequent doses of treatment (5 doses per day) that the participants were required to take as part of the trial, the crossover design, the long duration of the study (16 weeks of treatment), the strong taste of the black and green tea, and reports of teeth staining as mentioned by some participants.

Given the 51% attrition seen in this study, estimates of sample size for similar crossover tea studies would essentially double, and make the crossover design less efficient than the parallel design in our opinion. In addition, lack of a carryover effect in a parallel group design and more practical treatment administration, especially with multiple treatments, are other advantages of the parallel compared to the crossover trial design. Although the washout periods included in the crossover trials of tea are a safeguard against carryover effects, lack of knowledge on the intensity and duration of carryover effects limits the utility of the washout periods. Additionally, in most crossover trials, including the present study, washout periods between treatments are of the same duration thus assuming similar duration of carryover effects for all treatments in the trial which might not be true. For these reasons multi-arm chemoprevention trials of tea might be more practical and achieve better compliance if conducted using the parallel group design rather than the crossover design.

Efforts to make the treatments more palatable, either by providing the active ingredients in a pill or capsule form or by masking the taste of tea, might improve retention rates in tea trials. In addition, more research is needed to determine the dosage required for physiologic changes such that the strength of the tea and frequency of intake might be modified to ensure compliance in such trials. In this study participants were asked to drink five packets of tea every day. Since most participants were not habitual drinkers of tea, drinking five cups is a burden that might have resulted in the high percentage of dropouts seen in this study. In addition, participants were prescribed to drink tea using a “standard method” that asked them to hold the tea in the mouth for 30–60 seconds prior to swallowing. Although this method ensures standardization of dose delivery to oral cells for all participants it increases participant burden. Use of a placebo run-in period prior to randomization is another way to exclude participants who are less likely to be compliant over the period of the study. A short-term placebo run-in might be an effective way to decrease attrition and increase compliance in a parallel study, and also in a crossover study because the results from this study indicate that most of the dropouts occur after between the first and second treatments. However, participant exclusion based on a placebo run-in might limit the generalizability of the study results if participation and compliance are systematically associated with participant characteristics, such as, education, socioeconomic status, smoking, diet, and other behaviors. Crossover trials afford the advantage of having a participant as his/her own control in a different time period but multi-arm trials with assessment of more than two treatments might make the trial lengthy and increase participant burden.

Strengths of this study include conduct of a four-arm crossover trial of tea that has not been previously reported. In addition, data on participant characteristics, compliance, and dropout was accurately measured throughout the trial. However, this study has limitations. Although the four treatments were designed to be as similar to each other as possible in taste and color, it might not have led to 100% success with participant blinding because participants previously exposed to green or black tea could have easily identified the tea treatment. In addition, although some participants reported the cause of non-compliance and dropout from the study, a formal attempt was not made to determine the cause of dropout of all participants who left the study early.

## Conclusions

In summary, a high rate of attrition, especially among smokers, was observed in a crossover tea trial in smokers and non-smokers without premalignant oral lesions. Further research is needed to identify factors that mediate the observation of high dropout and low compliance seen in smokers on tea treatments in clinical trials. Clinical trials of tea with more than two treatments should consider the use of the parallel design rather than the crossover design to decrease participant burden and increase compliance.

## Competing interests

CD, JPR, EG, KM, KN, BD, FLC, LA-C have no competing interests to declare. YH was an employee of a private company (Tea solutions, Inc.) involved in tea processing during the conduct of this study

## Authors’ contributions

CD performed the statistical analysis and drafted the manuscript. F-LC conceived of the study, and participated in its design and coordination and helped to draft the manuscript. JPR helped in the data management and the analysis. EG helped in data management and assisted in drafting the manuscript. KM assisted with statistical analysis and reviewed the statistical sections of the draft. YH provided expertise on the tea treatments used in the study. KN and BD performed oral examinations on study participants, participated in study design and coordination, and provided clinical expertise for the manuscript. LLA-C participated in the study design and coordination and helped to draft the manuscript. All authors read and approved the final manuscript.

## Pre-publication history

The pre-publication history for this paper can be accessed here:

http://www.biomedcentral.com/1472-6882/12/96/prepub
